# Harnessing microbial communities for enhanced plant resilience against diseases

**DOI:** 10.3389/fmicb.2024.1500029

**Published:** 2025-04-11

**Authors:** Abdel Moneim E. Sulieman, Meshari Al-azmi, Naimah Asid Alanazi, Ahmed Eisa Ghoniem, Mohamed El-Sayed Hasan, Norah S. Alothman, Ayshah Alrashidi

**Affiliations:** 1 Department of Biology, College of Science, University of Ha'il, Ha'il, Saudi Arabia; 2 College of Computer Science and Engineering, University of Ha'il, Ha'il, Saudi Arabia; 3 Genetic Engineering and Biotechnology Research Institute (GEBRI), University of Sadat City, Sadat City, Egypt; 4 Biology Department, College of Science, University of Jazan, Jazan, Saudi Arabia

**Keywords:** biocontrol agents, disease management, integrated strategies, microbial consortia, *P. infestans*, plant resistance, *Pseudomonas* strains

## Abstract

**Background:**

*Phytophthora infestans* (*P. infestans*) and other plant infections threaten global agriculture and food security. This research incorporated *Pseudomonas* strains in microbial consortia to boost plant tolerance to *P. infestans*. The *P. infestans* fungus causes collapse and deterioration in many crops like potatoes by quickly spreading through their tubers and leaves in warm, damp weather.

**Objective:**

The main goals were to identify effective *Pseudomonas* strains (those with high inhibitory activity), test their interactions (both inhibitory and synergistic), and determine the effect of inoculum density on disease treatment.

**Methods:**

We used the following methodologies, from potato shoots and rhizosphere samples, Nine different strains of the antifungal bacterium *Pseudomonas* which were identified with preliminary antifungal activity. Bintje showed the greatest resistance to *P. infestans* among the three potato types that were examined. Methods utilized comprised: Quantification of bacterial density and growth, the inhibitory assays for *P. infestans*, experiments on leaf disc infections, Assessing the severity of an infection, Analysis of zoospore discharge. Studies on the integrated development of bacteria and valuation using statistical methods.

**Results:**

The study revealed the complexity of microbial interactions, host-specific reactions, and cell density's impact on treatment success. The study suggests using *Pseudomonas* strains as biocontrol agents, advancing sustainable agriculture. Microbial consortia disease management requires advanced methodologies, according to the findings. Investigating long-term ecological impacts on soil health, microbial diversity, and crop yield sustainability; validating identified microbial consortia through field trials; evaluating scalability and economic viability; and researching genetic engineering for customized disease control are recommended.

**Conclusions:**

Results suggest a shift from chemical pesticides to environmentally friendly plant disease control considering its ethical and regulatory implications. This study emphasizes the intricacy of microbial interactions and the need for informed biocontrol decisions. Their study also increases ecological knowledge and encourages innovative, sustainable worldwide agriculture.

## Introduction

Plant diseases pose a considerable risk to global agriculture, impacting food security, crop yields, and ecological equilibrium (Afridi et al., 2022). These microscopic adversaries, including several kinds of nematodes, fungi, viruses, and bacteria, take advantage of holes in plant defenses to cause diseases that can potentially wipe out entire crops (Verma et al., 2023). Unchecked plant diseases have far-reaching effects on human societies and the agricultural landscape (Firoozi and Firoozi, 2023). Plant pathogens result in significant financial losses due to decreased crop quality, lower yields, and higher production costs needed to treat these diseases (Ponnampalam and Holman, 2023). Beyond the immediate loss of crops, there is also an economic cost that includes managing diseases, controlling pests, and developing resistant crop varieties (Balaska et al., 2023).

Moreover, plant diseases have an ecological impact because they upset the ecosystem's delicate balance (Iqbal et al., 2023). As per the findings by (Ankit et al. 2020), the extensive application of chemical pesticides and fungicides in the fight against pathogens can cause environmental deterioration and have detrimental effects on non-target organisms, water quality, and soil health. Certain plant species' extinctions may occasionally have a domino effect on entire ecosystems (Sanders et al., 2018). Therefore, developing sustainable agricultural practices that balance environmental stewardship and productivity requires understanding plant pathogens and their complex effects (Kumawat et al., 2022). As one addresses modern agricultural challenges synthetic, it becomes more and more important to deal with the problems that plant pathogens present. This is essential for developing resilient, environmentally responsible, and commercially successful food production systems.

In plant pathology, *P. infestans* is a dangerous foe that primarily attacks potato plants, wreaking havoc on agriculture (Ivanov et al., 2021; Verma et al., 2023). The late blight disease, which has had a significant impact on global food security and left its mark on history, is caused by this infamouoomycete pathogen. Significant historical events,

Sprinkle P. plagues the mid-nineteenth-century Irish Potato Famine (Carpenter, 2015). Famine, relocation, and death confirmed late blight's severity (Goss et al., 2014). The pathogen's rapid evolution, versatility, and ability to wipe out potato harvests threaten global potato agriculture (Ramakrishnan et al., 2015). Years after the Irish Potato Famine, *P. infestans* damages crops (Corredor-Moreno and Saunders, 2019). National outbreaks have caused financial losses, food shortages, and livelihood disturbances (Workie et al., 2020). The historical and current significance of *P. infestans* highlights the necessity for appropriate strategies to mitigate its impact (Ortíz, 2023). Studying this illness is essential to ensuring the world's food supply and potato crops' resilience to biological threats (Botta et al., 2022).

Significant costs to human health, the economy, and the environment have resulted from the growing use of synthetic fertilizers to meet the world's food demands (Behera et al., 2021). In response to these challenges, microbial consortia, including *Pseudomonas* strains, have emerged as a promising alternative. According to (Morales-Cedeño et al. 2021) and (Rojas-Solís et al. 2020), plant growth-promoting bacteria (PGPB) are now crucial for plant development, soil fertility, and environmental health. The commercialization of PGPB as microbial inoculants has been prompted by their functional traits, which govern crop growth, development, and productivity (De los Santos Villalobos et al., 2018; Gupta et al., 2013). Microbial consortia are becoming increasingly popular as an environmentally friendly food production strategy to enhance the benefits offered by individual bacterial strains (Panwar et al., 2014; Poszytek et al., 2016; Sarma et al., 2015). Microbial consortia, which are often made up of two or more suitable bacteria interacting synergistically or additively, show higher activity levels than individual strains (Ju et al., 2019). According to (Villa-Rodríguez et al. 2019), these consortiums have exhibited remarkable efficacy in alleviating various stressors, such as pests, phytopathogenic infections, salinity, drought, and crop nutrient shortages.

Additionally, certain bacterial consortia can change nutrients, chelate iron, fix nitrogen in the soil, and produce phytohormones. These skills enhance soil quality and reduce the detrimental impacts of traditional farming methods (Gosal and Kaur, 2017). Based on the fermentation strategies used in the production of inoculants, simple and complex bacterial consortia can be distinguished from one another (Bashan et al., 2020). Under field conditions, these consortia's effectiveness depends on the strains' functionality and selection, which calls for consideration of factors like survival, post-inoculation persistence in the soil, and adaptation to unfavorable climatic conditions (Verbruggen et al., 2012).

In the context of microbial consortia, the strain selection process depends on the current environmental conditions in which these strains are intended to be applied, taking host, soil type, and climate into consideration. Remarkably, within a bacterial consortium, each strain serves as a functional rival to encourage plant growth in addition to complementing the others for plant and soil establishment (Morriën, 2016; Ney et al., 2018; Niu et al., 2020; Pandey et al., 2012). The complex interactions among bacterial strains in consortiums demonstrate the potential of these microbial communities as powerful tools for sustainable agriculture, offering the prospect of effective and environmentally friendly plant disease control.

In plant disease management, *Pseudomonas* strains are essential because of their exceptional capacity to suppress plant pathogens (David et al., 2018). Certain types within the *Pseudomonas* genus are recognized for their efficacy as biocontrol agents, attributed to their antagonistic characteristics and versatile metabolic capabilities (Mehmood et al., 2023). *Pseudomonas* strains can hinder the growth and development of various plant pathogens by producing antimicrobial compounds, including phenazines, pyrrolnitrin, and cyclic lipopeptides (Omoboye et al., 2019). Furthermore, these strains have successfully strengthened plants' built-in defenses against invasive pathogens by fostering systemic resistance (Shahid et al., 2021). Some strains of *Pseudomonas* are useful against *Phytophthora infestans* because they produce antibiotics, have siderophores, form biofilms, induce systemic resistance (ISR), and are compatible with farming methods. These bacteria are able to halt the progression of the infection because they produce antibiotics such as DAPG, phenazines, and pyrrolnitrin. Additionally, they inhibit the pathogen's growth by secreting siderophores, which trap iron. Strong biocontrol agents include *Pseudomonas* strains that can grow biofilms on the surfaces of roots (Santoyo et al., 2016).

Beyond simple pathogen inhibition, *Pseudomonas* strains effectively control disease (Haas and Keel, 2003). They are resilient parts of the plant's microbiome because of their adaptable nature, enabling them to flourish in various settings (Noman et al., 2021). *Pseudomonas* strains reduce the environment's ability to establish pathogens through competitive exclusion and niche occupation (Dutt et al., 2021). These strains' versatility, capacity to inhabit plant surfaces, and generation of secondary metabolites that provide resistance against phytopathogens all highlight their efficacy (Rieusset et al., 2020). *Pseudomonas* strains inhibit late blight pathogen *P. infestans* through different methods, making them useful in sustainable agriculture. These bacteria directly limit pathogen growth with antimicrobial substances including pyoluteorin and DAPG (Haas and Défago, 2005). In addition, they boost plant systemic resistance to infection (Pieterse et al., 2014). *Pseudomonas* competes for iron, limiting pathogen access and illness spread (Compant et al., 2005). By blocking pathogens, biofilm and root colonization protect plants (Lugtenberg and Kamilova, 2009). By improving nutrient availability, *Pseudomonas* boosts plant development and resilience (Bhattacharyya and Jha, 2012).

Therefore, using the biocontrol agent of *Pseudomonas* strains is a promising approach to the goal of environmentally friendly and sustainable plant disease management (Waghunde and Sabalpara, 2021). The research aims to enhance plant resistance to *P. infestans* using microbial consortia and environmentally friendly techniques. It assesses the effectiveness of *Pseudomonas* strains in suppressing the pathogen using dual culture Petri dish assays and leaf disk infection experiments. This multidisciplinary approach promotes sustainable plant disease management.

## Materials and methods

### Strain selection and identification

A selection of *Pseudomonas* strains was initially extracted from the rhizosphere (R) and shoot (S) regions of potato plants cultivated under field circumstances. A comprehensive identification and screening process was done to select strains with the most promising biocontrol agent. These strains were selected due to their superior inhibitory activity against *P. infestans* in preliminary tests. The approach commenced with the isolation of strains from rhizosphere soil and plant tissue samples, thereafter, cultured them on nutrient-specific media to facilitate *Pseudomonas* growth and enable preliminary morphological screening. Isolates were subsequently evaluated for antagonistic activity against *P. infestans in vitro* by dual-culture tests, wherein the capacity of each strain to suppress *P. infestans* growth was measured.

- Identification Procedure Morphological and Biochemical Assessment: Each isolate underwent Gram staining, oxidase and catalase tests, and growth on King's B agar to verify it identifies as belonging to the *Pseudomonas* genus. Morphological characteristics, including colony dimensions, pigmentation, and texture, were documented to aid in first classification.
- Molecular Identification: To precisely identify the species of each promising isolate, DNA was taken from bacterial cultures and subsequently subjected to 16S rRNA gene sequencing. Amplified sequences were compared to established *Pseudomonas* sequences in the NCBI GenBank database to verify species-level identification (Guyer et al., 2015; De Vrieze et al., 2020).
- Functional Screening for Biocontrol Agent: The nine strains identified for their robust *in vitro* inhibition of *P. infestans* were subsequently evaluated for biocontrol-related characteristics, including siderophore synthesis, protease activity, and hydrogen cyanide generation, as these factors are recognized for their antifungal efficacy.
- Final Selection: Nine strains were selected for future investigation based on their consistent and considerable inhibitory effects against *P. infestans* as demonstrated in these functional tests.

### Culture medium preparation

LB medium was primarily used for bacterial growth, while PIA medium facilitated the selective isolation of *Pseudomonas* strains. To prepare LB medium, 20 g of L^−1^ Difco LB broth (Lennox, USA) and 15 g of L^−1^ agar (Agar, ERNE Surface AG, Switzerland) were dissolved in distilled water. This standard procedure is used to obtain test bacterial cultures. After 2 days, cultivation was initiated by suspending individual colonies from the plates in 0.9% NaCl solution. Therefore, these suspensions were plated on LB agar medium as part of the culture procedure. This process creates a suspension of bacterial cells.

### Bacterial growth and density measurement

Bacteria were cultured overnight at 20°C and resuspended in 0.9% NaCl solution. (Guyer et al. 2015) performed bacterial competition experiments with derived strains resistant to rifampicin and nystatin. The experimental medium used in these studies contained Luria Bertani (LB) and *Pseudomonas* isolation agar (PIA) with or without the addition of rifampicin (50 mg/ml^−1^) and nystatin (500,000 U L^−1^) which were used to prevent contamination.

*Pseudomonas* isolation agar medium was prepared by dissolving 45 g of Fluka (*Pseudomonas* isolation agar per liter) in distilled water, then adding 20 ml of Sigma-Aldrich glycerol per liter. Optical density (OD) measurement at 570 nm was utilized to evaluate and regulate bacterial density. Although there were minor discrepancies in the cell count per OD_570_ unit among the nine strains, these variations consistently remained within a narrow range. With an OD_570_ of 1, most strains have a cell count between 1.4 × 10^8^ and 2 × 10^8^ cells per ml. In contrast, R32, S34, and S35 showed higher cell counts ranging from 4.5 × 10^8^ to 5.5 × 10^8^ cells per ml with an OD_570_ of 1.

### *P. infestans* inhibition tests

The *P. infestans* isolate Rec01 strain, which H. Krebs of Agroscope first isolated, was used in the inhibitory assays carried out in this investigation. Unclarified V8 (10%) media was used to culture the isolate to make sporangia and zoospore collection easier. In addition, experiments were carried out with the cultivation of fungi in pea medium. A crude B8 medium was prepared according to Miller's (1955) method by diluting B8 juice (100 ml L^−1^) and adding CaCO_3_ (1 g L^−1^) and 15 g L^−1^ of agar. V8 medium facilitates sporangia production, while pea agar provides a suitable growth substrate for the pathogen.

120 g of frozen peas were dissolved in an autoclave with water to create pea agar. The medium composition was then completed by adding 15 g of agar to the extracted grains. Potato slices were often sprayed with *P. infestans* isolates to provide movement to the host. Sporangium suspensions were prepared by scraping fungal tissue from 14-day-old plates and dispersing them in deionized water. Fungal tissue was removed from the suspension by filtering it through a cloth after thoroughly mixing it. A blank chamber was used to measure the concentration of spores, and the resultant suspension was kept in the dark until needed. To stop animal sporangia, the cold shock method was applied to sporangia. This was accomplished by transferring the spore suspension to eppendorf tubes filled with ice water and incubating it for 2 h at 4°C. to encourage the release of zoospores, the tubes were left to stand at room temperature for 20 min.

### Leaf disc infection experiment

Seven weeks after emergence, greenhouse-grown potato plants were harvested from the third and fourth leaves of Bintje, Lady Claire, and Victoria varieties. The different potato cultivars were chosen due to their varying levels of resistance to *P. infestans* and their different genetic backgrounds. These differences can impact disease development and response to pathogens, providing insight into the effectiveness of potential treatments across diverse genetic profiles. Using a cork cutter, leaf discs with a diameter of 1.8 cm were cut and put face up on a plate containing 1% water agar. Subsequently, 10 ml of a mixture containing bacterial suspension and spores was deposited in the center of each leaf disc. The final concentration of *P. infestans* was 125,000 sporangia/ml, with an OD_570_ of 0.9 for a single strain, 0.45 for a two-strain combination, and 0.3 for a three-strain combination. Instead of a negative control bacterial culture plate, 0.9% NaCl was used. The prepared boxes were then stored in an environment conducive to the growth of *P. infestans*, characterized by a temperature of 18°C, high humidity, and a lack of light.

### Evaluation of infection severity

After 7 days of incubation, domes formed. Infection severity was assessed by counting fruiting body maturity using ImageJ macros, as defined in a previous study by Guyer, De Vrieze, Bönisch, Gloor, Musa and Bodenhausen (2015).

Five replicates of each treatment were tested in two experiments with Lady Claire and Victoria. Each replicate contained five leaves from different plants, ensuring a complete and representative estimate. In contrast, 10 replicates were used for the cultivar Bintje, each consisting of 10 leaf discs from 10 independent plants. More devices available can increase the number of iterations. The difference in the number of replicates in Bintjes could be due to the greater number of plants available for this species compared to Lady Claire and Victoria. The evaluation included the effectiveness of 27 different treatments for each of the five plant groups. The evaluation involved comparing the infection levels of treated and untreated leaves of the same plant to measure the effect of different treatments.

The infection level of untreated control leaves was set to 100% for easy comparison. Afterward, the proportion of treated leaf discs with respect to the control group indicated the relative infection extent. The efficacy of the treatment is assessed using the following formula:


$$\begin{array}{l} Treatment\;Efficiency\;\left(\%\right)=100\\ \;-\;\left(Relative\;Infection\;Severity\;of\;Treatment\right) \end{array}$$ (1)

In this calculation, 0% represents the same level of infection as the untreated control group, while a treatment success rate of 100% indicates no infection. Negative values for the treated slide indicate a higher infection rate than for the untreated slide.

### Pea agar plate inoculation

Pea agar plates were inoculated simultaneously with bacterial strains and *P. infestans* seedlings. Subsequently, 10 ml of each bacterial suspension, prepared as described above, was consistently added, with three drops positioned at 10 mm from the dish's edge. 5 mm-diameter seedlings from a 14-day-old *P. infestans* culture were placed at the center of the dish. A negative control plate was also prepared by substituting the bacterial cell solution with 10 ml of 0.9% NaCl.

Three sets of plates, which were prepared in advance, were employed for the experiments. Following a 6-day incubation period in the absence of light at a temperature of 18°C, the plates were captured in photographs. Measuring the growth area of *P. infestans* seedlings with ImageJ is part of tissue growth assessment. The calculation of seedling relative growth involved dividing the measured cylindrical surface area on the plates treated with bacteria by the cylindrical surface area measured on negative control plates where no bacteria were present. This provides a relative measure of the effect of treatment on seedling growth. Treatment efficiency is then calculated using the formula above (100 – relative linear growth). The freshly prepared sporangia suspension was mixed with the bacterial suspension, followed by cold shock. 30 ml of the mixture was added to each well of a 24-well plate (Costar), and one well was treated. Images were then captured using a Cytation5 microplate reader (Biotek, USA) at 4 × magnification. After placing the animal tracks, take the first picture and then shake the board to spread the animal tracks evenly for later counting. Each treatment's average was calculated by summing the outcomes of the three experiments, which were conducted three times in total. This method provides a reliable and representative estimate of the test results.

### Zoospore release assessment

The relative zoospore release was determined by dividing the total number of zoospores released in treated samples by the average number of zoospores released in untreated samples. This method enables us to measure the impact of interventions on zoospore release in comparison to natural settings. A negative control of 0.9% NaCl was employed to determine the baseline for the inherent level of infection or background response. This control is essential for differentiating the impact of the pathogen treatment from any intrinsic plant responses.

Zoospore quantifications were performed to precisely measure *P. infestans* in the inoculum. Two methodologies were employed to guarantee accuracy: (1) manual enumeration via a hemocytometer, wherein zoospores are microscopically counted inside specified grid sections, and (2) automatic enumeration via image analysis software, which identifies and counts zoospores based on their dimensions and morphology. Both methodologies were utilized to mitigate human error and optimize the process, guaranteeing a precise evaluation of infection impacts.

The treatment effect, determined as 100% minus the relative buffer release, offered a quantitative assessment of the treatments' impact on zoospore release in comparison to the control.

Furthermore, each bacterial strain was examined both individually and in conjunction to assess growth and survival dynamics. This comprehensive approach was designed to investigate the potential impact of strain interactions on bacterial growth.

### Integrated bacterial development study

A better understanding of bacterial resilience in competitive situations was gained by studying ramp-resistant strains. Research on microbial adaptations on plant surfaces was enriched by observing interactions between resistant strains and wild-type strains. This observational study informed studies on competition and survival strategies. Here are the steps:

- A 0.45% NaCl solution was used to mix ramp-resistant and wild-type bacterial cultures at similar densities.
- The concoctions were left undisturbed in an incubator maintained at 18°C.
- Bacterial cells in a 0.9% NaCl solution were mixed with *P. infestans* sporangia in water in the same proportions as the quantities utilized in the leaf disc experiments. This particular concentration of NaCl was used to guarantee that the interactions between sporangia and bacteria were in line with those seen in trials conducted on leaf discs.
- At 1 and 5 day intervals, 1 ml of bacterial culture was mixed with 9 ml of fresh 0.9% NaCl solution to create 10-fold serial dilutions. These dilutions made it easier to count individual colonies and compare different species by reducing the bacterial density.
- Selected medium that might distinguish between species based on colony morphology, growth capacity, or rifampicin resistance were treated with 15 ml of each diluted sample.
- After incubation for 2 or 3 days, depending on the medium, CFUs/ml were tallied. By focusing on particular growth characteristics, selective media made it possible to differentiate between ramp-resistant and wild-type strains.

To gain a better understanding of the interactions and competition between different bacterial strains throughout time, counting CFUs/ml allowed for accurate quantification of each strain.

### Statistical analysis

Statistical analyses were performed using R software (version X.X), following the methods described by Sasaki et al. (2005). When appropriate, one- or two-way ANOVA was used comparing means and Dunnett's Tukey's for *post hoc* analysis, supported by the agricolae and multicomp techniques.

## Results

**Figure 1:**
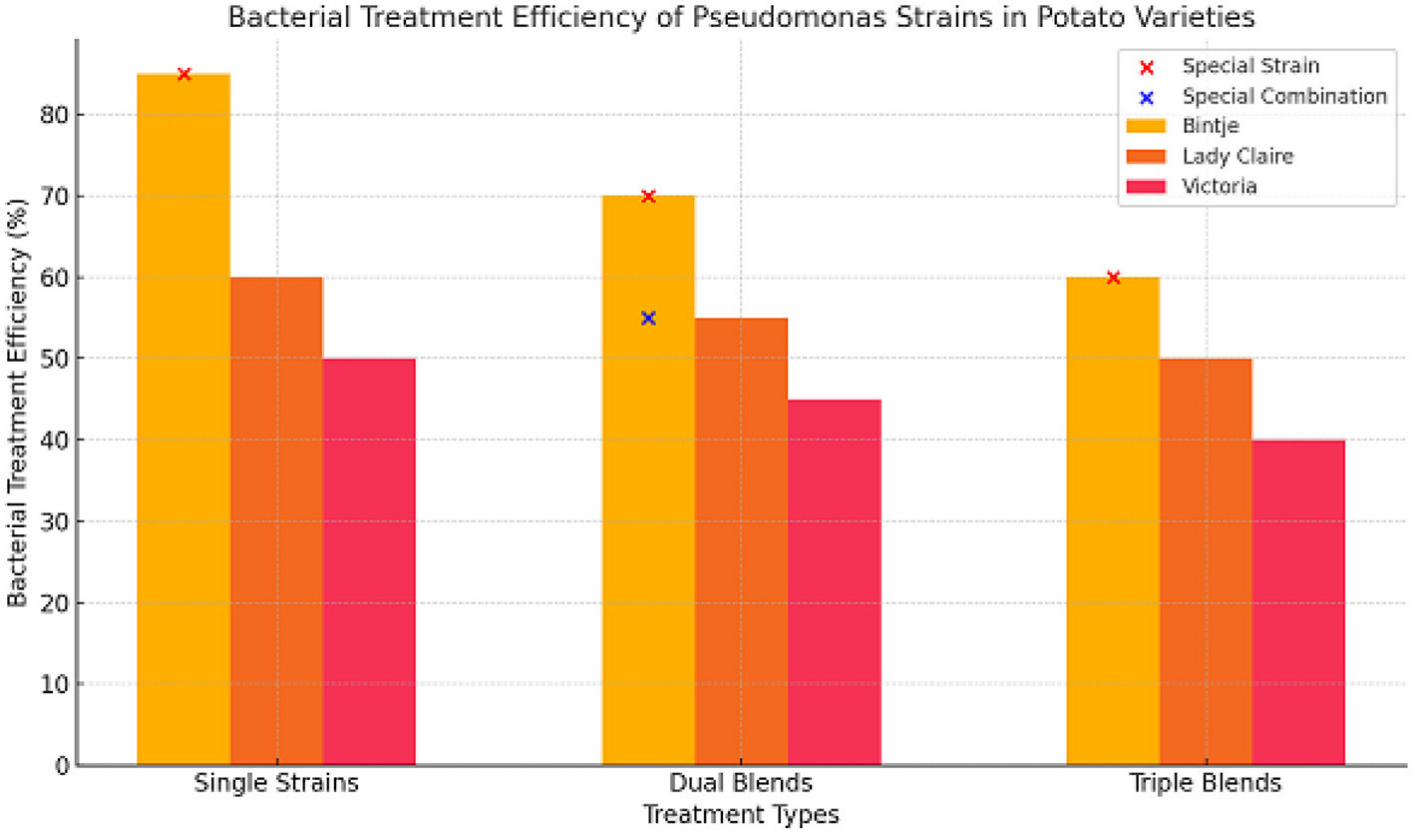
The relative bacterial treatment efficiency strains of *Pseudomonas* against *P. infestans* is evaluated on potato leaf discs. The effectiveness of the average of nine distinct strains of *Pseudomonas* in decreasing infections caused by *Phytophthora infestans* on leaf discs of three different potato varieties is illustrated in this image. An untreated control is used as a comparison, and each bar reflects the relative reduction in infection that was observed. The findings suggest that, on the whole, bacterial treatment was able to dramatically lower infection rates; however, there were some instances in which the treatment was unsuccessful. Because there is a possibility of interactions between potato cultivars and bacterial strains, independent studies were carried out for each cultivar in order to precisely evaluate the effectiveness of the treatment and the effects that were particular to various strains. The figure outlines the selection process for unique strains and special combinations of *Pseudomonas* strains, based on their demonstrated effectiveness in reducing *P. infestans* infections. These strains were chosen for their superior antifungal properties, and the combinations with the highest observed efficacy were designated as “special.”

In this experiment, nine *Pseudomonas* strains were selected in the phyllosphere (S) or rhizosphere (R) of potatoes grown in the field. *In vitro*, these strains showed varying degrees of antifungal activity. The main objective was to find out if using these strains together would provide better protection than using them separately. To achieve this goal, an experiment was conducted to infected leaf discs with 129 treatments. Includes single-use nine strains, plus 36 dual blends, and 84 triple blends. Three different potato varieties were used, each with a different susceptibility to late blight. Based on our choices, Binji is considered very sensitive, Lady Claire is considered sensitive, and Victoria is somewhat tolerant. As presented in the graphic (Figure 1) illustrates the efficacy of bacterial treatments across three categories: single strains, dual blends, and triple blends, for three potato varieties—Bintje, Lady Claire, and Victoria.

- Single Strains: The maximum treatment efficacy was recorded in Bintje (~85%), followed by Lady Claire (~60%) and Victoria (~50%). Among individual strains, only S35 significantly diminished the rate of illness development across all kinds, highlighting its potential as an effective treatment. Conversely, R84 exhibited minimal activity, underscoring the diversity in strain performance.
- Dual Blends: A moderate enhancement in efficiency was observed in some combinations. Dual blends such as S04/S49, S19/S49, R32/S34, R76/S49, R84/S35, and R84/S49 demonstrated considerable efficacy across all potato cultivars, achieving about 70% for Bintje, 55% for Lady Claire, and 45% for Victoria. Only six out of 36 combinations provided protection for all types, showing that strain pairing must be conducted strategically.
- Triple Blends: The efficiency diminished relative to dual blends (~60% for Bintje, ~50% for Lady Claire, and ~40% for Victoria), indicating possible antagonistic interactions across strains in triple blends.

The results show that different types of bacteria, different potato varieties, and different mixing strategies have different effects on the effectiveness of bacterial treatment:

- Potato Varieties: In terms of overall response to bacterial treatments, Bintje had the highest level of sensitivity to late blight, which may explain why there were more noticeable variances across the varieties. Victoria, on the other hand, was slightly tolerant but showed the least improvement in efficiency, which is consistent with its natural resistance mechanisms.
- Effects on Specific Strains: Its broad-spectrum antifungal activity is highlighted by S35's outstanding effectiveness across all types. On the flip side, R84's inertness highlights how crucial strain selection is when developing efficient treatments.
- A Variety of Treatment Options: In several instances, dual blends were more effective than single strains, lending credence to the idea that certain combinations (such as S04/S49) can boost efficiency through synergy. Nevertheless, it appears that there may be incompatibility among the various strains due to the decreased effectiveness of triple blends.

**Figure 2:**
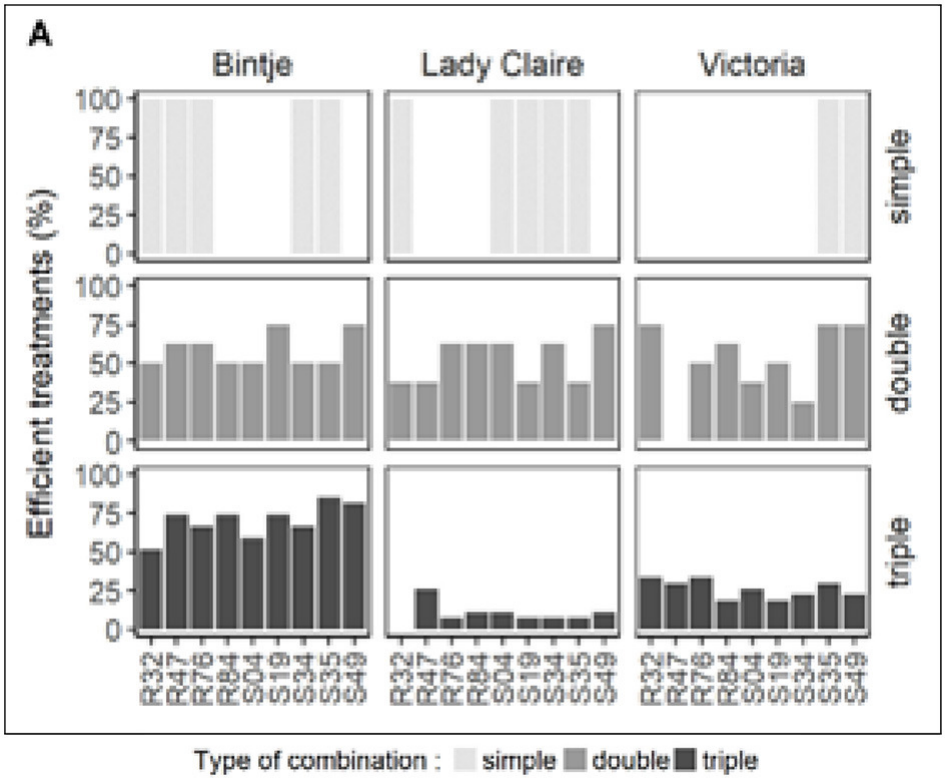
*Pseudomonas* strains' relative bacterial treatment efficiency on potato leaf discs that were infected with *Phytophthora infestans*, this figure depicts the relative effectiveness of *Pseudomonas* strains that were treated in single, double, and triple combinations in suppressing the growth of sporophores. The effectiveness of treatments was determined by whether or not they significantly reduced the development of sporophores in comparison to the controls. For each bacterial treatment, the figure comprises two critical measures, which are as follows: (1) the percentage of successful treatments that achieved a significant reduction in the development of sporophores, and (2) the percentage of treatments in which each particular bacterium contributed to the effectiveness that was seen.

To understand the strains' efficacy in single, double, and triple combinations, we computed the percentage of effective treatments. The criteria are based on treatments that have significantly reduced disease symptoms for all three options. As shown in Figure 2, this test was performed for each strain by the respective route of administration, alone or in combination. Strain diversity significantly affects the relative efficacy of strains under different application methods. However, some strains showed consistent changes between the three methods. For example, when R84 was combined with one or two other strains, I always had better results than when used alone.

For S49, Bintje and Dame Claire showed consistent trends, showing better results when the strains were used together than when they were used individually. Specifically, when it came to therapy success rates, Bintje strains were often more effective in triple combinations than in double combinations. As shown in Figure 2, Lady Claire and Victoria show opposite trends. The relationship between the lower percentage of successful treatments and changes in inoculum density in the Lady Claire and Victoria triple combinations remains unclear. This uncertainty is due to differences in bacterial cell density observed between single, double, and triple treatments: OD of 0.9 for single treatment, 0.45 for double treatment, and 0.3 for triple treatment. To investigate the protective effect of this unique strain at three different seeding densities, experiments were conducted to answer this question.

**Figure 3:**
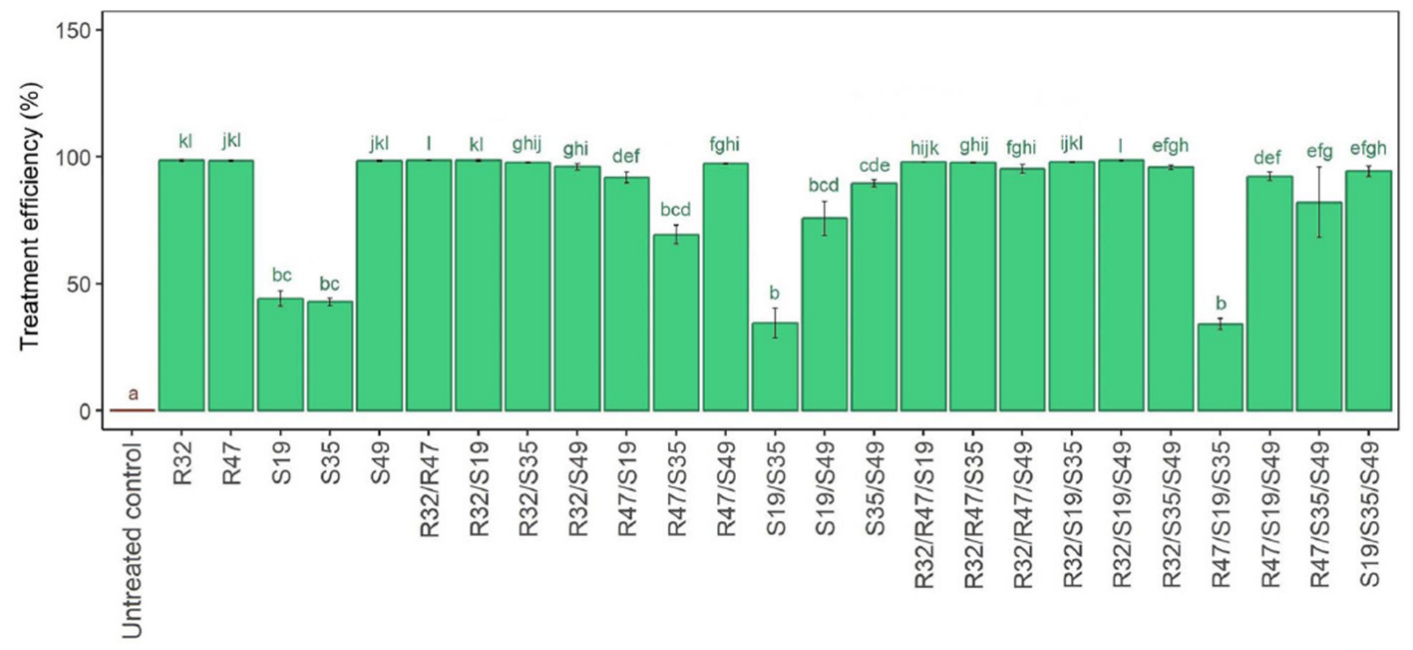
Relative bacterial treatment efficiency of *Pseudomonas* strains' hyphal growth suppression in single, double, and triple combinations with *P. infestans*. Five *Pseudomona* strains were treated singly, in pairs, and in triplet combinations in a duplicate culture experiment, as shown in this image, which shows the relative inhibition of *Phytophthora infestans* hyphal development. A 100-fold decrease in the relative growth area of *P. infestans* in comparison to the control was considered treatment effectiveness. The small letters above the bars in the figure indicate statistically significant differences between treatments, as determined by the Kruskal-Wallis test. Treatments with the same letter are not significantly different from each other, while treatments with different letters show significant differences in their effects on the hyphal growth of *Phytophthora infestans*.

A single injection of the studied strains (R32, R47, and S49) resulted in total suppression of *P. infestans* hyphal development. Conversely, strains S19 and S35 had a less severe but notable growth suppression. Contrary to predictions, combining two “weaker” strains unexpectedly did not result in a greater suppression of filamentous development. Furthermore, as shown in Figure 3, the “strongest” strain retained its inhibitory power even when combined with other effective strains. An interesting observation was made when S19 or S35 was mixed with R32, which resulted in the same complete inhibition of fungal growth as when R32 was administered alone.

R47 and S49 work differently when connected to S19 or S35. Its effect is weak, particularly in the pairings R47/S35 and S49/S19 and somewhat in the S35/S49 pair. For ternary combinations, the addition of R32 is beneficial because it confers strong activity on many base pairs, covering the S19/S35 combination, which was inactive at first, as well as the R47/S35 and S19/S49 combinations, which were a little bit inactive (Figure 3). However, the putatively “inactive” S19/S35 linker did not become more effective in inhibiting inhibition when active R47 was added.

Derived from the findings of this experiment, one can infer that (i) R32 will be the most effective food additive in double and triple formulations; (ii) S49 improves efficiency only in the S19/S35 scenario but not in other positive-effect double combinations; (iii) R47 typically has very little beneficial effect in the triple combination and can only slightly increase the effectiveness of S19/S35 (Figure 3).

In the flap disc test and this experiment, all voltages were applied at an optical density of 0.9. At the same time, the double combination had a lower optical density of 0.45, and the triple combination had an optical density of 0.3. A study was conducted to determine if a change in the number of cells at the beginning of the experiment affected the degree of growth inhibition. Surprisingly, none of the studied strains showed such an inhibitory effect on seedling growth.

**Figure 4:**
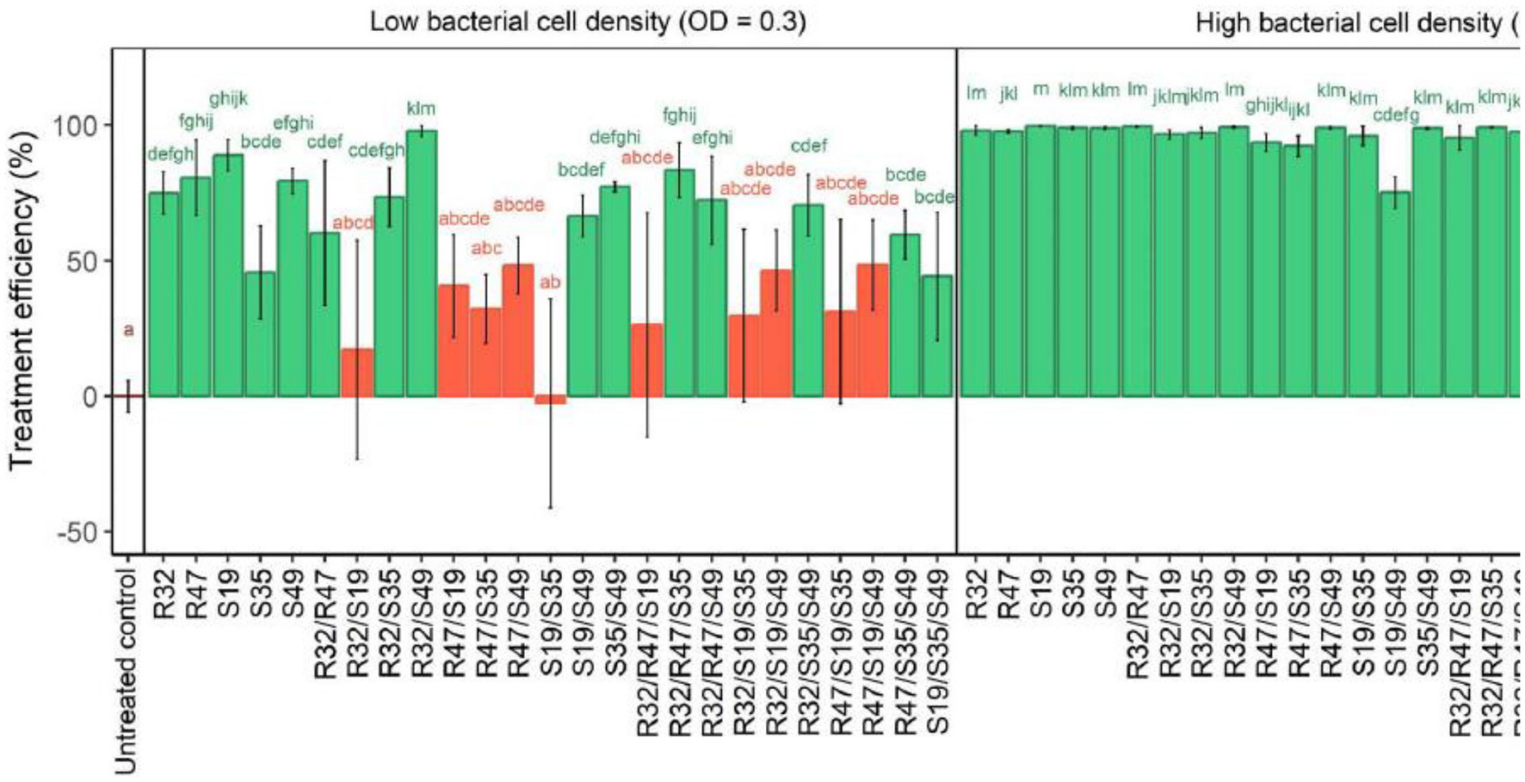
Relative efficiency of cell density in *Pseudomonas* strain on the release of *P. infestans* zoospores. The impact of five different *Pseudomonas* strains' cell densities on the release of *Phytophthora infestans* zoospores is depicted in this figure. A 100-fold decrease in zoospore yield in comparison to untreated controls was considered treatment success. Each cell density was investigated for its effect on lowering zoospore formation, and their effects on zoospore suppression were examined. The small letters above the bars in the figure indicate statistically significant differences between treatments, as determined by the Kruskal-Wallis test. Treatments with the same letter are not significantly different from each other, while treatments with different letters show significant differences in their effects on the hyphal growth of *Phytophthora infestans*.

Preliminary experiments indicate that the overall cell density of a strain plays an important role in its efficacy against *P. infestans* zoospores, whether used alone or in combination. As a result, two distinct optical densities were used for the analysis: a greater optical density (OD = 0.9) and a lower optical density (OD = 0.3). All treatments showed significant inhibition of zoospores when bacteria were added at OD 0.9 (see Figure 4). In particular, the dual combination S19/S49 showed less reduced zoospores release than each strain administered alone. However, the reduction was significant compared to the reference. A more dilute application (OD = 0.3) showed decreased activity, particularly in the case of S19 combined with S35, R32, or R47, resulting in a complete loss of activity. The notable activity shown when the strains were treated alone was not restored when the various strains were added to S19/R32, S19/S35, or S19/R47 (Figure 4).

The figure shows how different *Pseudomonas* strain combinations suppress *P. infestans* zoospore discharge at varying cell densities. Low bacterial cell densities had little or no efficacy, while high densities reduced zoospore generation, with most strain combinations being 100% efficient. Disparities decreased with greater cell density, suggesting strain heterogeneity was corrected. Negative *y*-axis values indicate that treated samples produced more zoospores than controls, suggesting incomplete cell density or strain selection can increase pathogen activity.

Experiments with lower cell densities showed synergism, contrasting previously reported contrasts between S19 and other strains. While one strain allowed ~20% of the spores to release zoospores, the combination of S49 and R32 almost completely inhibited the release of zoospores. Similarly, S49 and S35 inhibited the release of zoospores more consistently (less variable) than each strain did alone. Not only did this pair (S35/S49) show a large reduction in population, but so did all other pairs. On the other hand, compared with R47 (4/10) and particularly S19 (2/10), exhibited reduced activity. After considering the 10 potential double and triple combinations, the selection was reached by evaluating the overall number of successful combinations for each strain. S49 showed the highest activity (7/10), followed by S35 and R32 (6/10). This indicates that R32, S35, and S49 favorably impacted the other strains in their mixture while being tested in the zoo. In contrast, R47 and especially S19 hurt the same strain (see Figure 4).

**Figure 5:**
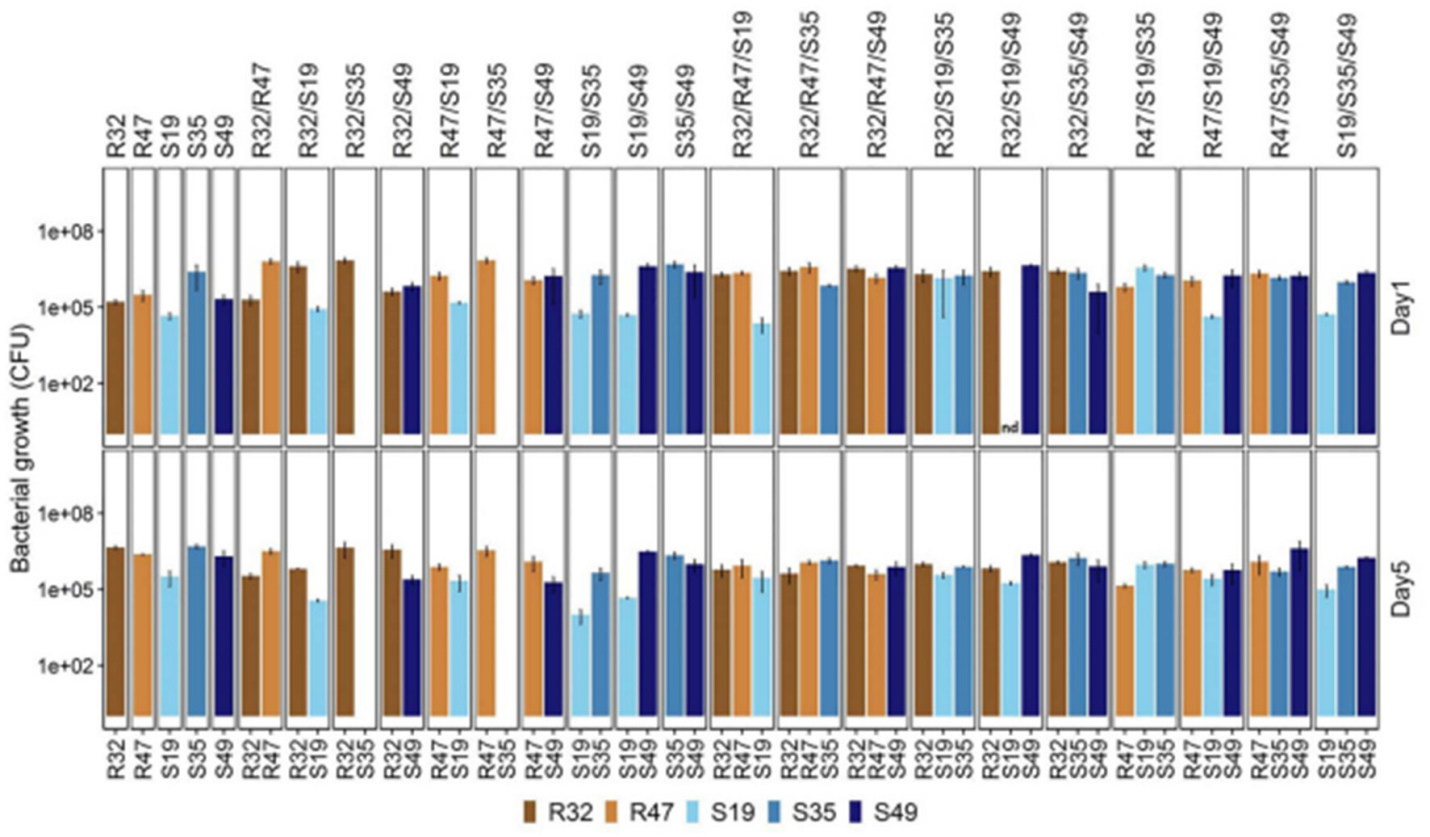
To evaluate the survival and persistence of various *Pseudomonas* strains, their numbers were measured in a 0.9% NaCl solution. At each stage of the incubation process, which lasted between 1 and 5 days, the strains' abundance was measured. Each strain's maintained viability—or capacity to endure in saline circumstances over time—was verified by a 5-day measurement. This assessment sheds light on the stability and possible efficacy of each strain when used in comparable environmental settings.

Some of the above data suggest that direct stimulatory or inhibitory effects may exist between strains. Five selected strains were grown in a mixture of duplicates and triplicates in saline solutions for 5 days to see if cultivation under conditions identical to those used in the leaf disk assay resulted in survival or preferential growth of specific strains. At the end of the experiment and every 2 days, the relative abundance of each strain was measured. It should be noted that the initial total cell density (as determined by optical density) for each treatment in this experiment was the same. Thus, each strain starts with half the inoculum compared to a single vaccination. Still, it contains a third of the inoculum in double and triple combinations instead of treating individual infections. Despite this initial difference, the total number of colony-forming units (CFU) at the end of the experiment (all strain combinations considered) was significantly higher for the double and triple combinations than for the single inoculations (see Figure 5). This suggests that even without a nutrient source in the saline culture medium, (i) The strain can offset the reduced primary cell density throughout the 5-day growth period, and (ii) the strain generally does not make sacrifices. Growth comes from the other.

After 5 days, four infected strains individually reached densities of ~1 million colony-forming units per milliliter (CFU/ml). Even in a nutrient-poor environment, their numbers decreased slightly every other day and showed a moderate increase from the first to the fifth day. After the first day, the amount of S19 was less than that of other species. Over the next 4 days, stem development was limited or even reduced in abundance, depending on the composition (see Figure 5 and Table 1).

**Table 1:** Characteristics of five selected *Pseudomonas* strains.

**stress**	**The origin of isolation**	**Food science**	**Hydrogen cyanide**	**Phenazine**
P32	Rhizosphere	Stench	Yes	No
P47	Rhizosphere	Chlorophyll	Yes	Yes
C19	Circle of leaves	Frederiksbergen	No	No
S35	Circle of leaves	Fluorescence	No	No
S49	Circle of leaves	Fluorescence	Yes	No

A captivating observation about S35 was made. When grown alongside two rhizosphere strains, R32 and R47, it seems to be completely suppressed or even eradicated, in contrast to its usual decline in abundance when coexisting with other strains. Remarkably, S35 does not appear to have any beneficial effects on these latter species. This is evident because their quantity does not increase when S35 is present compared to when it is not, as illustrated in Figure 5. The inhibitory effect of S35 is reversed with the addition of each extra strain when in the presence of R32 or R47. Consequently, S35 exhibits regular maturation in all studied triple combinations, including R32 and R47.

Table 2 shows the counting of CFU/ml for ramp-resistant and wild-type strains at 1 and 5 days post-incubation. Selective media were used to distinguish strains by growth and resistance.

**Table 2:** Comparison of CFU/ml for ramp-resistant and wild-type strains at 1 and 5 days.

**Bacterial strain**	**Day 1 CFU/ml**	**Day 5 CFU/ml**	**Activity**
Ramp-resistant strain A	1.2 × 10^6^	2.8 × 10^7^	Significant increase, indicating resilience and effective adaptation in competitive situations.
Ramp-resistant strain B	9.5 × 10^5^	1.7 × 10^6^	Moderate growth observed, maintaining presence in competitive conditions.
Wild-type strain A	1.5 × 10^6^	3.0 × 10^6^	Initial growth was strong but showed limited increase by Day 5, indicating competition effects.
Wild-type strain B	1.1 × 10^6^	2.5 × 10^6^	Stable growth pattern but lower than ramp-resistant counterparts, suggesting competitive disadvantage.

Patterns of Growth: By Day 5, the ramp-resistant strains had significantly increased their CFU/ml, showing that they could tolerate and even thrive in the highly competitive environment. On the other hand, ramp-resistant mutants showed rapid expansion while wild-type bacteria grew only moderately. On Day 1, both the ramp-resistant and wild-type strains grew similarly. However, by Day 5, the wild-type strains' growth had stalled, demonstrating the long-term endurance and competitive advantage of the ramp-resistant strains.

For the mechanisms of adaptation, the disparities in growth rates could be explained by different ways ramp-resistant strains have evolved to thrive in environments that are both competitive and potentially stressful. These adaptations could include things like improved nutrient uptake efficiency or resistance to antimicrobial chemicals.

The significance of comprehending microbial adaptations in agricultural and ecological settings is highlighted by our findings, which offer light on the competitive interactions between ramp-resistant and wild-type bacterial strains.

## Discussion

### Introduction: addressing *P. infestans* through enhanced plant defense

This research focused on developing strategies to bolster plant defenses against *P. infestans*, a pathogen with devastating impacts on agriculture, both historically and in current farming practices. Due to the significant threat posed by this oomycete, effective management strategies are essential to protect crop health (Ivanov et al., 2021; Carpenter, 2015). The study explored the biocontrol agent of *Pseudomonas* strains and examined how their interactions within microbial consortia contribute to disease suppression.

### Aligning with sustainable agriculture goals

This investigation aligns with broader objectives in sustainable agriculture, aiming to foster environmentally friendly approaches to disease management (Morriën, 2016; Ney et al., 2018; Pandey et al., 2012). *Pseudomonas* strains, known for their adaptability and ability to inhibit a range of plant pathogens, were chosen for their suitability in integrated biocontrol strategies (David et al., 2018). By analyzing interactions between *Pseudomonas* strains and *P. infestans*, the study addresses a critical gap in research and enhances our understanding of plant-microbe interactions.

### Microbial consortia: a shift toward environmentally responsible farming

The application of bacterial consortia to control phytopathogens reflects a shift toward sustainable agriculture. Research shows that microbial communities can work synergistically to benefit crops, thus supporting the use of consortia in disease management (Villa-Rodríguez et al., 2019). This aligns with the global movement toward reducing chemical inputs in agriculture, where microbial consortia are seen as essential tools for enhancing crop resilience and controlling pathogens in an eco-friendly manner (Bashan et al., 2020).

### Antifungal activities of *Pseudomonas* strains

The study's experiments, including leaf disc infection tests and dual-culture Petri dish assays, revealed varying antifungal activities among the nine *Pseudomonas* strains tested. Certain strains, such as S35 and S49, demonstrated strong, consistent protection against *P. infestans*. Additionally, the use of triple strain combinations yielded promising results, with some combinations showing enhanced resistance in various potato varieties. Notably, strain S35 underscored the complexity of microbial interactions, as it exhibited synergistic effects when combined with other strains.

### Host-specific responses and tailored biocontrol strategies

The study found that the protective efficacy of *Pseudomonas* strains varied among different potato varieties, highlighting the importance of host-specific strategies in biocontrol. These findings emphasize the need for tailoring biocontrol approaches to the specific plant host, ensuring optimized results for disease suppression.

### Inoculum density and microbial dynamics

A crucial factor in the efficacy of biocontrol was the density of the bacterial inoculum. The study observed that different starting cell densities affected strain efficacy, underscoring the importance of understanding microbial population dynamics for successful real-world applications. The influence of *Pseudomonas* strains on zoospore release from *P. infestans* also provided valuable insights into how microbial interactions might impact pathogen reproduction.

### Significance of integrated strategies and future directions

This research highlights the complexity of microbial consortia in disease control and the need for integrated strategies that leverage specific strain contributions (Ju et al., 2019). By identifying the unique roles of *Pseudomonas* strains in microbial consortia, the study supports the potential of these strains in advancing sustainable agriculture. This knowledge is crucial for developing robust, eco-friendly disease management practices that ensure long-term agricultural productivity and ecosystem health.

### Comparison of CFU/ml for ramp-resistant and wild-type strains

The observed growth patterns indicate that ramp-resistant bacterial strains demonstrate a significant competitive advantage over wild-type strains in similar environments, as evidenced by their marked increase in CFU/ml by Day 5. While both strains exhibited comparable initial growth, the wild-type strains' growth stagnated, revealing their inability to sustain competitive performance over time. This discrepancy underscores the resilience of ramp-resistant strains, likely attributable to their adaptive mechanisms, such as enhanced nutrient uptake efficiency and resistance to antimicrobial compounds. These findings highlight the critical role of understanding microbial adaptations in agricultural and ecological contexts, as they provide valuable insights into the dynamics of microbial communities and their potential applications in biocontrol strategies.

### Implications of the study

This study's implications go beyond the lab, providing useful information for creating environmentally friendly and long-lasting plant disease management techniques. The potential for developing efficient biocontrol agents against *P. infestans*, a pathogen recognized for its past and present effects on potato cultivation worldwide, is provided by the synergistic effects of *Pseudomonas* strains in integrated microbial consortia, as noted by (Ortíz 2023). Farmers and other agricultural practitioners may be able to lessen their dependency on chemical pesticides and lessen the environmental damage caused by their widespread use by utilizing the biocontrol agent of particular *Pseudomonas* strains (Ankit et al., 2020; Gosal and Kaur, 2017; Panwar et al., 2014). A possible shift toward more targeted and sustainable approaches is suggested by the demonstrated effectiveness of certain strains and their combinations in suppressing late blight disease. This aligns with the global imperative for ecologically responsible food production (Gupta et al., 2013; Villa-Rodríguez et al., 2019). The use of microbial consortia, particularly in triple combinations, demonstrated enhanced efficacy over single-strain treatments, suggesting the importance of synergistic effects in “biocontrol.”

### Limitations of the study

It is crucial to recognize the study's limitations in order to comprehend its context and breadth. The effectiveness of *Pseudomonas* strains against a wider variety of pathogens has not been investigated, but studies have mostly concentrated on their biocontrol agent against *P. infestans*. To expand their use for new crops, future research should examine the interactions between *Pseudomonas* strains and various plant diseases.

The dependence on controlled surroundings and *in vitro* experiments is a major drawback because they do not completely mimic field circumstances. Soil moisture, temperature, and pH can vary greatly, for example, and this can have a major impact on microbial activity and interactions; however, these variables are not present in *in vitro* circumstances. Laboratory settings do not take soil and climate heterogeneity into consideration, which affects the survival of microbes and the effectiveness of biocontrol. It is essential to expose microbial consortiums to these natural variations in field trials in order to evaluate the robustness and adaptability of *Pseudomonas* strains.

Furthermore, there are operational and logistical hurdles associated with the scalability of microbial consortiums for large-scale farming. It may be challenging to reliably distribute appropriate microbial concentrations across large areas in field applications, particularly when dealing with unpredictable weather and different soil types. Additionally, considerations including the expenses associated with manufacturing, storing, and transporting microbial inoculants on a commercial scale need to be taken into account. These biocontrol tactics might be improved and validated through long-term field research that include various agricultural systems, soil types, climate zones, and crop rotations. This would guarantee that they are feasible for extensive application in agriculture (Gosal and Kaur, 2017).

### Future research recommendations

This study explores the use of microbial communities for plant disease resilience, highlighting the potential of reducing reliance on chemical pesticides and promoting natural plant defenses. It suggests that beneficial strains like *Pseudomonas* can enhance crop resilience across various types. The research suggests practical implementation through bio-inoculants, soil amendments, or seed treatments, contributing to long-term agricultural sustainability.

Studying the identified microbial consortia's scalability and practical applicability is important for future research. It's important to consider the economic viability and the ease of combining these biocontrol techniques with current agricultural practices. For biocontrol agents to be widely used in different farming systems, this evaluation is crucial. Also, we need studies that follow *Pseudomonas* strains over time to see how they affect soil health, microbial diversity, and sustainable crop yields, as well as how these factors interact with repeated applications.

It is also crucial to investigate the molecular mechanisms behind the different strain efficacy and synergistic effects in greater depth. Investigating the hereditary components of these relationships may provide light on where to focus efforts to improve biocontrol efficiency. One way to improve *Pseudomonas* strains' biocontrol capabilities is to genetically engineer them with desirable properties, such as increased resistance to environmental stresses, better colonization capacity, or antifungal enzyme production.

Cooperation between agricultural scientists and microbiologists is essential to help get research from the lab to the field. Collaborations in different fields could make it easier to adapt microbial consortia to real-world situations and speed up the process of developing scaled application approaches. More effective microbial inoculants might be created with the help of this collaborative approach, which could also lead to targeted genetic improvements that are in line with sustainable farming techniques.

## Conclusion

In conclusion, our study successfully demonstrated the potential of *Pseudomonas* strains in enhancing plant resistance to *P. infestans*. Notably, strains S35 and S49 exhibited significant antifungal activity, consistently providing protection against the pathogen. The promising results from the triple combinations further underscore the potential of these biocontrol agents. This research advocates for a reduced reliance on chemical pesticides, aligning with the principles of sustainable agriculture and offering practical implications for farmers.

However, several limitations must be addressed, including the necessity for extensive field testing and a thorough financial viability analysis to ensure practical implementation. Future research should focus on genetic engineering to enhance biocontrol traits, as well as exploring scalability and real-world applications of these findings. By promoting the use of *Pseudomonas* strains as part of integrated pest management programs, this research paves the way for more sustainable agricultural practices, potentially reducing dependency on chemical pesticides and minimizing their environmental impact.

## Data Availability

Study data are included in the article; further inquiries may be directed to the corresponding author.

## References

[B1] Afridi M. S. Ali S. Salam A. K. Terra W. C. Hafeez A. Sumaira . (2022). Plant microbiome engineering: hopes or hypes. Biology 11:1782. doi: 10.3390/biology11121782 36552290 PMC9774975

[B2] Ankit Saha L. Kishor V. Bauddh K. (2020). “Impacts of synthetic pesticides on soil health and non-targeted Flora and Fauna,” in Ecological and Practical Applications for Sustainable Agriculture, eds. K. Bauddh, S., Kumar, R., Singh, and J., Korstad (Singapore: Springer), 65–88. doi: 10.1007/978-981-15-3372-3_4

[B3] Balaska V. Adamidou Z. Vryzas Z. Gasteratos A. (2023). Sustainable crop protection via robotics and artificial intelligence solutions. Machines 11:774. doi: 10.3390/machines11080774

[B4] Bashan Y. Prabhu S. R. de-Bashan L. E. Kloepper J. W. (2020). Disclosure of exact fermentation protocols, identity of microorganisms within consortia, and formation of advanced consortia with microbe-based products. Biol. Fertil. Soils 56, 443–445. doi: 10.1007/s00374-020-01464-x

[B5] Behera B. Das T. P. Raj R. Ghosh S. Raza B. Sen S. (2021). Microbial consortia for sustaining productivity of non-legume crops: prospects and challenges. Agric. Res. 10, 1–14. doi: 10.1007/s40003-020-00482-3

[B6] Bhattacharyya P. N. Jha D. K. (2012). Plant growth-promoting rhizobacteria (PGPR): emergence in agriculture. World J. Microbiol. Biotechnol. 28, 1327–1350. 22805914 10.1007/s11274-011-0979-9

[B7] Botta A. Cavallone P. Baglieri L. Colucci G. Tagliavini L. Quaglia G. (2022). A review of robots, perception, and tasks in precision agriculture. Appl. Mech. 3, 830–854. doi: 10.3390/applmech3030049

[B8] Carpenter P. A. M. (2015). Redressing the silence: Photography, memory and the Great Famine (Doctoral diss). Curtin University.

[B9] Compant S. Duffy B. Nowak J. Clément C. Barka E. A. (2005). Use of plant growth-promoting bacteria for biocontrol of plant diseases: principles, mechanisms of action, and future prospects. Appl. Environ. Microbiol. 71, 4951–4959. 16151072 10.1128/AEM.71.9.4951-4959.2005 PMC1214602

[B10] Corredor-Moreno P. Saunders D. G. O. (2019). Expecting the unexpected: factors influencing the emergence of fungal and oomycete plant pathogens. New Phytol. 225, 118–125. doi: 10.1111/nph.16007 31225901 PMC6916378

[B11] David B. V. Chandrasehar G. Selvam P. N. (2018). “*Pseudomonas fluorescens*: a plant-growth-promoting rhizobacterium (PGPR) with potential role in biocontrol of pests of crops,” in Crop Improvement Through Microbial Biotechnology, eds. R. Prasad, S. S. Gill, and N. Tuteja (Amsterdam: Elsevier), 221–243. doi: 10.1016/B978-0-444-63987-5.00010-4

[B12] De los Santos Villalobos S. Parra Cota F. I. Herrera Sepúlveda A. Valenzuela Aragón B. Estrada Mora J. C. (2018). Colmena: colección de microorganismos edáficos y endófitos nativos, para contribuir a la seguridad alimentaria nacional. Rev. Mexicana Ciencias Agrícolas 9, 191–202. doi: 10.29312/remexca.v9i1.858

[B13] De Vrieze M. Varadarajan A. R. Schneeberger K. Bailly A. Rohr R. P. Ahrens C. H. . (2020). Linking comparative genomics of nine potato-associated *Pseudomonas* isolates with their differing biocontrol potential against late blight. Front. Microbiol. 11:857. doi: 10.3389/fmicb.2020.00857 32425922 PMC7204214

[B14] Dutt A. Andrivon D. Le May C. (2021). Multi-infections, competitive interactions, and pathogen coexistence. Plant Pathol. 71, 5–22. doi: 10.1111/ppa.13469

[B15] Firoozi A. Firoozi A. A. (2023). Eco-friendly materials for contaminated ground remediation: a study on the efficacy of plant fibers, green stones, and anti-bacterial substances. New Environ. Friend. Mater. 2, 1–10. doi: 10.55121/nefm.v2i2.107

[B16] Gosal S. K. Kaur J. (2017). “Microbial inoculants: a novel approach for better plant microbiome interactions,” in Probiotics in Agroecosystem, eds. V. Kumar, M. Kumar, S. Sharma, and R. Prasad (Singapore: Springer), 269–289. doi: 10.1007/978-981-10-4059-7_14

[B17] Goss E. M. Tabima J. F. Cooke D. E. L. Restrepo S. Fry W. E. Forbes G. A. . (2014). The Irish potato famine pathogen *P. infestans* originated in Central Mexico rather than the Andes. Proc. Natl. Acad. Sci. U.S.A. 111, 8791–8796. doi: 10.1073/pnas.1401884111 24889615 PMC4066499

[B18] Gupta V. K. Schmoll M. Maki M. Tuohy M. Mazutti M. A. (2013). Applications of Microbial Engineering. Boca Raton, FL: CRC Press. doi: 10.1201/b15250

[B19] Guyer A. De Vrieze M. Bönisch D. Gloor R. Musa T. Bodenhausen N. . (2015). The anti-phytophthora effect of selected potato-associated *Pseudomonas* strains: from the laboratory to the field. Front. Microbiol. 6:1309. doi: 10.3389/fmicb.2015.01309 26640460 PMC4661289

[B20] Haas D. Défago G. (2005). Biological control of soil-borne pathogens by fluorescent pseudomonads. Nat. Rev. Microbiol. 3, 307–319. 15759041 10.1038/nrmicro1129

[B21] Haas D. Keel C. (2003). Regulation ofantibioticproduction inroot-colonizing *Pseudomonas* spp. and relevance for biological control of plant disease. Annu. Rev. Phytopathol. 41, 117–153. doi: 10.1146/annurev.phyto.41.052002.095656 12730389

[B22] Iqbal B. Li G. Alabbosh K. F. Hussain H. Khan I. Tariq M. . (2023). Advancing environmental sustainability through microbial reprogramming in growth improvement, stress alleviation, and phytoremediation. Plant Stress 10:100283. doi: 10.1016/j.stress.2023.100283

[B23] Ivanov A. A. Ukladov E. O. Golubeva T. S. (2021). *Phytophthora infestans*: an overview of methods and attempts to combat late blight. J. Fungi 7:1071. doi: 10.3390/jof7121071 34947053 PMC8707485

[B24] Ju W. Liu L. Fang L. Cui Y. Duan C. Wu H. (2019). Impact of co-inoculation with plant-growth-promoting rhizobacteria and rhizobium on the biochemical responses of alfalfa-soil system in copper contaminated soil. Ecotoxicol. Environ. Saf. 167, 218–226. doi: 10.1016/j.ecoenv.2018.10.016 30342354

[B25] Kumawat K. C. Razdan N. Saharan K. (2022). Rhizospheric microbiome: bio-based emerging strategies for sustainable agriculture development and future perspectives. Microbiol. Res. 254:126901. doi: 10.1016/j.micres.2021.126901 34700186

[B26] Lugtenberg B. Kamilova F. (2009). Plant-growth-promoting rhizobacteria. Annu. Rev. Phytopathol. 63, 541–556. 19575558 10.1146/annurev.micro.62.081307.162918

[B27] Mehmood N. Saeed M. Zafarullah S. Hyder S. Rizvi Z. F. Gondal A. S. . (2023). Multifaceted impacts of plant-beneficial *Pseudomonas* spp. in managing various plant diseases and crop yield improvement. ACS Omega 8, 22296–22315. doi: 10.1021/acsomega.3c00870 37396244 PMC10308577

[B28] Miller P. M. (1955). V-8 juice agar as a general purpose medium for fungi and bacteria. Phytopathology 45, 461–462.

[B29] Morales-Cedeño L. R. del Carmen Orozco-Mosqueda M. Loeza-Lara P. D. Parra-Cota F. I. de los Santos-Villalobos S. Santoyo G. (2021). Plant growth-promoting bacterial endophytes as biocontrol agents of pre- and post-harvest diseases: fundamentals, methods of application and future perspectives. Microbiol. Res. 242:126612. doi: 10.1016/j.micres.2020.126612 33059112

[B30] Morriën E. (2016). Understanding soil food web dynamics, how close do we get? Soil Biol. Biochem. 102, 10–13. doi: 10.1016/j.soilbio.2016.06.022

[B31] Ney L. Franklin D. Mahmud K. Cabrera M. Hancock D. Habteselassie M. . (2018). Examining trophic-level nematode community structure and nitrogen mineralization to assess local effective microorganisms' role in nitrogen availability of swine effluent to forage crops. Appl. Soil Ecol. 130, 209–218. doi: 10.1016/j.apsoil.2018.06.015

[B32] Niu B. Wang W. Yuan Z. Sederoff R. R. Sederoff H. Chiang V. L. . (2020). Microbial interactions within multiple-strain biological control agents impact soil-borne plant disease. Front. Microbiol. 11:585404. doi: 10.3389/fmicb.2020.585404 33162962 PMC7581727

[B33] Noman M. Ahmed T. Ijaz U. Shahid M. Azizullah Li, D. . (2021). Plant–microbiome crosstalk: dawning from composition and assembly of microbial community to improvement of disease resilience in plants. Int. J. Mol. Sci. 22:6852. doi: 10.3390/ijms22136852 34202205 PMC8269294

[B34] Omoboye O. O. Oni F. E. Batool H. Yimer H. Z. De Mot R. Höfte M. (2019). Pseudomonas cyclic lipopeptides suppress the rice blast fungus *Magnaporthe oryzae* by induced resistance and direct antagonism. Front. Plant Sci. 10:901. doi: 10.3389/fpls.2019.00901 31354771 PMC6636606

[B35] Ortíz R. (2023). Challenges for crop improvement. Emerg. Top. Life Sci. 7, 197–205. doi: 10.1042/ETLS20230106 37905719

[B36] Pandey P. Bisht S. Sood A. Aeron A. Sharma G. Maheshwari D. K. (2012). “Consortium of plant-growth-promoting bacteria: future perspective in agriculture,” in Bacteria in Agrobiology: Plant Probiotics, ed. D. Maheshwari (Berlin: Springer EBooks), 185–200. doi: 10.1007/978-3-642-27515-9_10

[B37] Panwar M. Tewari R. Nayyar H. (2014). “Microbial consortium of plant growth-promoting rhizobacteria improves the performance of plants growing in stressed soils: an overview,” in Phosphate Solubilizing Microorganisms: Principles and Application of Microphos Technology, eds. M. Khan, A. Zaidi, and J. Musarrat (Cham: Springer), 257–285. doi: 10.1007/978-3-319-08216-5_11

[B38] Pieterse C. M. Zamioudis C. Berendsen R. L. Weller D. M. Van Wees S. C. Bakker P. A. (2014). Induced systemic resistance by beneficial microbes. Annu. Rev. Phytopathol. 52, 347–375. 24906124 10.1146/annurev-phyto-082712-102340

[B39] Ponnampalam E. N. Holman B. W. B. (2023). “Chapter 22 - Sustainability II: sustainable animal production and meat processing,” in Lawrie's Meat Science, ed. F. Toldrá (Sawston: Woodhead Publishing). doi: 10.1016/B978-0-323-85408-5.00001-7

[B40] Poszytek K. Cieżkowska M. Skłodowska A. Drewniak Ł. (2016). Microbial consortium with high cellulolytic activity (MCHCA) for enhanced biogas production. Front. Microbiol. 7:324. doi: 10.3389/fmicb.2016.00324 27014244 PMC4791528

[B41] Ramakrishnan A. P. Ritland C. E. Blas Sevillano R. H. Riseman A. (2015). Review of potato molecular markers to enhance trait selection. Am. J. Potato Res. 92, 455–472. doi: 10.1007/s12230-015-9455-7

[B42] Rieusset L. Rey M. Muller D. Vacheron J. Gerin F. Dubost A. . (2020). Secondary metabolites from plant-associated *Pseudomonas* are overproduced in biofilm. Microb. Biotechnol. 13, 1562–1580. doi: 10.1111/1751-7915.13598 33000552 PMC7415375

[B43] Rojas-Solís D. Vences-Guzmán M. Á. Sohlenkamp C. Santoyo G. (2020). Antifungal and plant growth–promoting Bacillus under saline stress modify their membrane composition. J. Soil Sci. Plant Nutr. 20, 1549–1559. doi: 10.1007/s42729-020-00246-6

[B44] Sanders D. Thébault E. Kehoe R. Frank van Veen F. J. (2018). Trophic redundancy reduces vulnerability to extinction cascades. Proc. Natl. Acad. Sci. U.S.A. 115, 2419–2424. doi: 10.1073/pnas.1716825115 29467292 PMC5878001

[B45] Santoyo G. Moreno-Hagelsieb G. Orozco-Mosqueda M. C. Glick B. R. (2016). Plant growth-promoting bacterial endophytes. Microbiol. Res. 183, 92–99. doi: 10.1016/j.micres.2015.11.008 26805622

[B46] Sarma B. K. Yadav S. K. Singh S. Singh H. B. (2015). Microbial consortium-mediated plant defense against phytopathogens: readdressing for enhancing efficacy. Soil Biol. Biochem. 87, 25–33. doi: 10.1016/j.soilbio.2015.04.001

[B47] Sasaki T.N Massaki Kubo T. (2005). Wolbachia variant that induces two distinct reproductive phenotypes in different hosts. Heredity 95, 389–393. doi: 10.1038/sj.hdy.6800737 16106260

[B48] Shahid I. Han J. Hardie D. Baig D. N. Malik K. A. Borchers C. H. . (2021). Profiling of antimicrobial metabolites of plant growth promoting *Pseudomonas* spp. isolated from different plant hosts. 3 Biotech 11, 1–14. doi: 10.1007/s13205-020-02585-8 33489669 PMC7801537

[B49] Verbruggen E. Heijden M. G. A. Rillig M. C. Kiers E. T. (2012). Mycorrhizal fungal establishment in agricultural soils: factors determining inoculation success. New Phytol. 197, 1104–1109. doi: 10.1111/j.1469-8137.2012.04348.x 23495389

[B50] Verma R. C. Amulothu D. V. R. T. Suhas P. D. Nain M. Kumar D. Saikanth D. R. K. . (2023). A comprehensive overview of plant pathology understanding disease mechanisms and control. Int. J. Plant Soil Sci. 35, 1207–1214. doi: 10.9734/ijpss/2023/v35i203918

[B51] Villa-Rodríguez E. Parra-Cota F. Castro-Longoria E. López-Cervantes J. de los Santos-Villalobos S. (2019). Bacillus subtilis TE3: a promising biological control agent against Bipolaris sorokiniana, the causal agent of spot blotch in wheat (*Triticum turgidum* L. subsp. durum). Biol. Control 132, 135–143. doi: 10.1016/j.biocontrol.2019.02.012

[B52] Waghunde R. R. Sabalpara A. N. (2021). Impact of *Pseudomonas* spp. on plant growth, lytic enzymes and secondary metabolites production. Front. Agron. 3:752196. doi: 10.3389/fagro.2021.752196

[B53] Workie E. Mackolil J. Nyika J. Ramadas S. (2020). Deciphering the impact of COVID-19 pandemic on food security, agriculture, and livelihoods: a review of the evidence from developing countries. Curr. Res. Environ. Sustain. 2:100014. doi: 10.1016/j.crsust.2020.100014 34977605 PMC7550095

