# Correction: Harnessing microbial communities for enhanced plant resilience against diseases

**DOI:** 10.3389/fmicb.2025.1714004

**Published:** 2025-11-17

**Authors:** Abdel Moneim E. Sulieman, Meshari Al-azmi, Naimah Asid Alanazi, Ahmed Eisa Ghoniem, Mohamed El-Sayed Hasan, Norah S. Alothman, Ayshah Alrashidi

**Affiliations:** 1 Department of Biology, College of Science, University of Ha'il, Ha'il, Saudi Arabia; 2 College of Computer Science and Engineering, University of Ha'il, Ha'il, Saudi Arabia; 3 Genetic Engineering and Biotechnology Research Institute (GEBRI), University of Sadat City, Sadat City, Egypt; 4 Biology Department, College of Science, University of Jazan, Jazan, Saudi Arabia

**Keywords:** biocontrol agents, disease management, integrated strategies, microbial consortia, *P. infestans*, plant resistance, *Pseudomonas* strains

Author Ahmed Said Mohamed Elnahal has requested to be removed from this published article. The correct author and affiliations list is as follows:

Abdel Moneim E. Suliema^1*^, Meshari Al-azmi^2^, Naimah Asid Alanazi^1^, Ahmed Eisa Ghoniem^1^, Mohamed El-Sayed Hasan^3^, Norah S. Alothman^4^, and Ayshah Alrashidi^1^.

^1^Department of Biology, College of Science, University of Ha'il, Ha'il, Saudi Arabia

^2^College of Computer Science and Engineering, University of Ha'il, Ha'il, Saudi Arabia

^3^Genetic Engineering and Biotechnology Research Institute (GEBRI), University of Sadat City, Sadat City, Egypt

^4^Biology Department, College of Science, University of Jazan, Jazan, Saudi Arabia

The original version of this article has been updated, including affiliations, copyright statement, author contributions, and the article citation, to reflect the removal of Ahmed Said Mohamed Elnahal.

